# Causal Exposures in Pancreatic Cancer Incidence: Insights From Mendelian Randomization Studies

**DOI:** 10.1002/jgh3.70105

**Published:** 2025-02-03

**Authors:** Ashraf Mohamadkhani, Reza Ghanbari, Ramin Shakeri, Mohammad Ali Mohammadkhani, Akram Pourshams

**Affiliations:** ^1^ Liver and Pancreatobiliary Diseases Research Center; Digestive Diseases Research Institute, Shariati Hospital, Tehran University of Medical Sciences Tehran Iran; ^2^ Gene Therapy Research Center, Digestive Diseases Research Institute, Tehran University of Medical Sciences Tehran Iran; ^3^ Digestive Oncology Research Center, Digestive Diseases Research Institute, Tehran University of Medical Sciences Tehran Iran; ^4^ Technical and Vocational University Tehran Iran

**Keywords:** causal factors, Mendelian randomization, pancreatic cancer

## Abstract

**Aim:**

Pancreatic cancer, marked by its high lethality and poor 5‐year survival rate, requires a thorough understanding of its risk factors and etiological mechanisms. In this review, we collected the latest findings from Mendelian randomization (MR) studies to identify potential causal factors for pancreatic cancer.

**Method and Results:**

The present analysis encompasses MR studies on the gut and oral microbiomes, non‐malignant phenotypes, blood metabolites, immune cells, and chronic inflammation. Specific gut and oral microbiome species have been identified as potential causal factors for pancreatic cancer, some with protective effects, and others increasing the risk. The review also highlights causal associations between obesity, type 2 diabetes, and pancreatic cancer, as well as the impact of blood metabolites and immune cell phenotypes on disease risk. Additionally, it investigates the causal effects of inflammatory bowel disease, showing a significant risk increase associated with Crohn's disease.

**Conclusion:**

These insights emphasize the need for interdisciplinary research and personalized medicine to enhance prevention and treatment strategies for pancreatic cancer.

## 1 Introduction

Pancreatic cancer is recognized as one of the aggressive malignancies within the gastrointestinal tract, characterized by its high lethality and a poor 5‐year survival rate of merely 10% [1]. Understanding the risk factors and etiological mechanisms associated with this disease is crucial for identifying susceptible populations and mitigating the overall the disease burden [2]. Current research indicates that the initiation and progression of pancreatic cancer are influenced by a combination of genetic mutations, dietary habits, and environmental factors [3, 4]. Recent advancements in metagenomic sequencing and 16S ribosomal RNA analysis have unveiled a significant association between gut microbiota and the development of pancreatic cancer [5]. This emerging perspective highlights the complexity of the disease's etiology, suggesting that both intrinsic and extrinsic factors contribute to its pathogenesis [6, 7]. The multifactorial nature of pancreatic cancer necessitates a comprehensive approach to research and prevention, focusing on both genetic predispositions and modifiable lifestyle factors. Understanding these elements can lead to improved screening strategies and potentially reduce the incidence and mortality associated with this formidable disease.

The complexity and diversity of microbial communities in the oral cavity, and gastrointestinal tract of patients with pancreatic cancer exhibit significant alterations when compared to those of healthy individuals [8, 9]. Dysbiosis of the gut microbiota not only has direct implications for gastrointestinal diseases but also extends its effects to extraintestinal organs, such as the pancreas and liver, potentially triggering an inflammatory response [5]. This inflammatory response may facilitate the reintroduction of microbiota into the pancreatic duct, a phenomenon supported by the increased incidence of pancreatic ductal adenocarcinoma (PDAC) observed in the head of the pancreas [10]. Furthermore, the complex interactions between oral pathogens and host immune responses, along with the metabolites they produce, play a role in tumorigenesis. These findings suggest a plausible link between oral microbial dysbiosis and the progression of cancer, underscoring the need for further investigation into the relationship between microbial health and cancer development [11, 12].

Body fat is also a significant modifiable risk factor for various cancers, which can be stored both subcutaneously and viscerally [13]. One notable phenomenon is intra‐pancreatic fat deposition (IPFD), which is hypothesized to contribute to the development of PDAC. Research indicates that individuals with elevated levels of IPFD exhibit impaired insulin secretion, potentially due to localized lipid signaling within pancreatic cells. This impairment may increase the risk of developing diabetes [14], further complicating the relationship between obesity and pancreatic cancer [14, 15].

Pancreatic cancers are often referred as “cold tumors” due to their expression of relatively few neoantigens that can be recognized by immune cells [16]. Nevertheless, chronic inflammatory diseases have been shown to increase the risk of cancer, leading to investigations into the relationship between inflammatory bowel disease (IBD) and pancreatic cancer. While some studies have reported a connection [17], a recent meta‐analysis did not find a significant association [18]. However, it is noteworthy that the risk of pancreatic cancer appears to be higher in IBD patients who also have primary sclerosing cholangitis [19]. Understanding the immune phenotype and exploring the relationship between immune‐related diseases such as IBD and pancreatic cancer may provide valuable insights for the clinical management of IBD. This knowledge could enhance active surveillance, facilitate early diagnosis, and support accurate clinical decision‐making for high‐risk subpopulations of pancreatic cancer [20].

Mendelian randomization (MR) is an analytical method that utilizes genetic variation associated with a risk factor as instrumental variables to determine whether a causal effect exists on outcomes, thereby minimizing the influence of confounding factors [21]. By examining correlational outcomes, MR enables the inference of potential causality and provides a more robust assessment of the relationship between exposure and outcome [22]. Leveraging MR to assess the causal status of modifiable exposures identified in previous studies could be instrumental in addressing the research gap related to the factors that contribute to pancreatic cancer. This approach may enhance early diagnosis and support accurate clinical decision‐making.

In the present comprehensive review, we analyzed and interpreted the latest findings from Mendelian Randomization (MR)‐based studies to explore potential causal factors associated with the incidence of pancreatic cancer.

## 2 Search Strategy

Currently, Mendelian randomization (MR) methods are increasingly being utilized to investigate the causal relationship between various factors, including the human microbiome, inflammatory states, and metabolic states, and their potential impact on pancreatic cancer [23]. By using genetic variants as instruments, MR studies aim to distinguish between correlation and causation, thereby reducing the influence of confounding variables and reverse causation that often complicate observational studies. This is particularly valuable because traditional epidemiological methods may struggle to establish clear causal relationships due to the presence of unobserved confounding factors. The use of MR in this area can provide more robust estimates of causal effects, contributing to a better understanding of the underlying mechanisms and potentially informing preventive and therapeutic strategies [24].

Here, we conducted a comprehensive review of published results from Mendelian randomization studies on pancreatic cancer up to June 2024. Our search included articles published in PubMed, MEDLINE (Ovid), EMBASE, and the Cochrane Library (Cochrane Central Register of Controlled Trials). We excluded published review articles and editorials from our analysis.

The search algorithm used was: (“Mendelian randomization” OR “genetic tool variable” OR), (“genome‐wide association studies” OR “GWAS”), AND (“pancreatic cancer” OR “pancreatic ductal adenocarcinoma” OR “PDAC”) and causality with no caption restrictions. All retrieved articles were thoroughly checked for relevant citations to ensure a comprehensive inclusion of pertinent studies. The majority of the articles identified were either review articles, unrelated to the causative risk factors for pancreatic cancer, or did not involve human studies. Ultimately, only 10 published articles remained that met our study's inclusion criteria and were evaluated.

The following sections highlight the key findings obtained from the investigation of the causal relationship between various exposure factors and the incidence of pancreatic cancer. These findings were comprehensively assessed through studies employing the Mendelian randomization approach. The findings from these MR studies offer valuable insights into the etiology and underlying mechanisms of pancreatic cancer, contributing to a deeper understanding of the disease's pathogenesis. This knowledge can be crucial for the development of preventive strategies and therapeutic interventions aimed at reducing the incidence and improving the outcomes of pancreatic cancer.

### 2.1 Causal Relationship Between Gut Microbiome and Pancreatic Cancer

In a two‐sample MR analysis using publicly available genome‐wide association studies (GWAS) data, researchers investigated the causal effects of gut bacteria on pancreatic cancer risk. This study identified six gut bacteria at the genus level that were causally associated with pancreatic cancer. However, “*Alloprevotella*” (OR: 0.752, 0.570–0.993, *p* = 0.045) was excluded due to its weak instrumental variable status. The analysis revealed that while “*Senegalimassilia*” (OR: 0.635, 0.403–0.049, *p* = 0.04) had a protective effect against pancreatic cancer, “*Odoribacter*” (OR: 1.899, 1.157–3.116, *p* = 0.011), “*Ruminiclostridium* 9” (OR: 1.976, 1.128–3.461, *p* = 0.017), “*Ruminococcaceae* (*UCG011*)” (OR: 1.433, 1.072–1.916, *p* = 0.015), and “*Streptococcus*” (OR:1.712, 1.071–1.736, *p* = 0.025) were found to be causative of pancreatic cancer. Sensitivity analysis indicated no evidence of heterogeneity, horizontal pleiotropy, or reverse causality between the gut bacteria and pancreatic cancer, suggesting a robust causal relationship between specific gut bacteria and the risk of pancreatic cancer. This study primarily employed the inverse variance weighted (IVW) method and validated the results through sensitivity analysis, Cochran's Q test, MR‐PRESSO, and MR‐Egger regression. These analyses revealed no evidence of heterogeneity, horizontal pleiotropy, or reverse causality between the gut microbiota and pancreatic cancer [5].

In another recent study, Li et al. investigated the causal relationship between gut microbiota and pancreatic cancer using MR and colocalization analysis, as well as identified potential genes involved in this mechanism. The study utilized a comprehensive dataset from the MiBioGen consortium, which included summaries of 211 gut microbiome types, combined with GWAS data from the National Human Genome Research Institute‐European Bioinformatics Institute (NHGRI‐EBI). Using three complementary MR methods, the researchers determined that nine gut microbiome variants were causally associated with pancreatic cancer. Among these, four gut microbiomes exhibited a protective effect, while five were associated with an increased risk of pancreatic cancer. Furthermore, colocalization analysis revealed that two genes, MCM6 and RPS26, were involved in the interaction between the gut microbiome and pancreatic cancer [10].

### 2.2 Investigation of Oral Microbiome as a Causal Factor for Pancreatic Cancer

Feng et al. conducted a study to explore the potential causality of the oral microbiome as an exposure factor for seven major cancers, including pancreatic cancer, the East Asian population. Given that previous research has indicated differences in oral microbiota composition between Asian populations and other ethnic groups, which could influence cancer initiation and progression, the study focused on data from GWAS studies conducted on East Asian populations for two‐sample MR.

For pancreatic cancer, the analysis identified a total of 48 bacterial species in the tongue (comprising 26 genera and 18 families) and 51 bacterial species in saliva (comprising 29 genera and 21 families). However, the results showed that *Fusobacterium* and Veillonellaceae F0422 emerged as potential risk factors, with odds ratios greater than 1.0. In contrast, several genera were found to confer protective benefits against pancreatic cancer. These included *Prevotella*, *Oribacterium*, *Aggregatibacter*, *Solobacterium*, *Pauljensenia*, *Streptococcus*, *Gemella*, *Porphyromonas*, *Saccharimonadaceae TM7x*, and *Lancefieldella*. These findings suggest that specific components of the oral microbiome may play a causal role in the risk and protection against pancreatic cancer (OR < 1) [25]. The study suggested that the inflammation‐promoting actions of the oral microbiome could serve as a universal pathway influencing cancer risk. This implies that dysbiosis, or an imbalance in the oral microbiota, may contribute to the development of various cancers through inflammatory mechanisms.

The causal effects of gut bacteria and bacterial species from tongue and saliva on pancreatic cancer were summarized in Table 1.

**TABLE 1:** Causal exposures in pancreatic cancer incidence.

Authors	GWAS data	Exposure factor	MR analysis OR (95% CI)	*p*
Jiang et al. 2023 [5]	MiBioGen FinnGen R9	*Senegalimassilia*	0.635 (0.403–0.998)	0.049
		*Odoribacter*	1.899 (1.157–3.116)	0.011
		*Ruminiclostridium 9*	1.976 (1.128–3.461)	0.017
		*Ruminococcaceae* (*UCG011*)	1.433 (1.072–1.916)	0.015
		*Streptococcus*	1.712 (1.071–1.736)	0.025
Li et al. 2024 [10]	MiBioGen NHGRI‐EBI	Family. Unknown family. Id. 1 000 001 214	1.53 (1.00 to 2.35)	0.048
			1.02 (0.31 to 3.24)	0.97
			1.26 (0.74–2.15)	
		Genus. *Parasutterella.id.2892*	0.53 (0.33–0.84)	0.007
			0.69 (0.18–2.67)	0.060
			0.45 (0.24–0.83)	0.011
		Genus. *LachnospiraceaeUCG004.id.11324*	0.49 (0.28–0.87)	0.015
			0.81 (0.09–7.53)	0.859
			0.59 (0.27–1.27)	0.177
		Genus. *unknowngenus.id.1000001215*	1.53 (1.00–2.35)	0.048
			1.02 (0.31–3.34)	0.97
			1.26 (0.72–2.20)	0.426
		Genus. *sutterella.id.2896*	2.45 (1.38–4.37)	0.002
			2.03 (0.13–30.86)	0.621
			2.54 (1.18–5.44)	0.017
		Genus. *Eggerthella.id.819*	0.63 (0.43–0.93)	0.02
			0.64 (0.11–3.84)	0.634
			0.59 (0.35–1.00)	0.05
		Order. *Gastranaerophilales.id.1591*	1.53 (1.00–2.35)	0.048
			1.02 (0.31–3.34)	0.97
			1.26 (0.71–2.21)	
		Genus. *Oxalobacter.id.2978*	0.69 (0.49–0.98)	0.035
			0.45 (0.09–2.21)	0.352
			0.68 (0.44–1.07)	0.094
		Class. *Melainabacteria.id.1589*	1.55 (1.03–2.32)	0.035
			1.07 (0.34–3.35)	0.913
			1.18 (0.70–1.98)	0.452
Feng, Ren, and Wang, 2023 [25]	East Asian populations	*Fusobacterium* and Veillonellaceae F0422	(OR > 1)	< 0.05
		*Prevotella*, *Oribacterium*, *Aggregatibacter*, *Solobacterium*, *Pauljensenia*, *Streptococcus*, *Gemella*, *Porphyromonas*, *Saccharimonadaceae TM7x*, and *Lancefieldella*	(OR < 1)	< 0.05
Yamazaki et al. 2024 [15]	UK Biobank PanScan I‐III PanC4	Intra‐pancreatic fat deposition	2.46 (1.38–4.40)	< 0.01
Maina et al. 2023 [26]	UK Biobank	Adiposity polygenic scores by calculating weighted polygenic scores (PGS) for BMI = PGSBMI	1.0804 (1.025–1.14)	0.0037
		PGSBMI after adjustment for type two diabetes	1.073 (1.018–1.13)	0.00904
		PGS for BMI‐adjusted waist‐hip ratio (WHRadjBMI) = PGSWHRadjBMI	1.047 (0.99–1.104)	0.086
		PGSWHRadjBMI after adjustment for type 2 diabetes	1.039 (0.99–1.097)	0.14
		WHRadjBMI	1.00095 (1.00011–1.0018)	0.027
King et al. 2023 [27]	PanScan PanC4	Nonalcoholic fatty liver disease	1.04 (0.88–1.22)	< 0.05
			0.89 (0.65–1.21)	
			1.07 (0.90–1.27)	
			0.93 (0.67–1.28)	
Tan et al. 2024 [14]	PanScan I‐III, PanC4 and BioVU	*PPIP5K2* gene	1.587 (1.257, 2.005)	0.0001
		*TFR2* gene	1.755 (1.344, 2.290)	0.00003
		*HNF4G* gene	1.906 (1.372, 2.648)	0.0001
		*LRRC10B* gene	0.543 (0.400, 0.737)	0.00008
		*PRC1* gene	7.389 (2.690, 20.302)	0.0001
		*FBXL20* gene	0.241 (0.121, 0.480)	0.00005
		*INHBA* gene	0.573 (0.463, 0.710)	0.0000003
		*SMC2* gene	1.448 (1.274, 1.645)	0.00000001
		*ABO* gene	1.147 (1.094, 1.203)	0.00000001
		*PDX1* gene	0.476 (0.386, 0.587)	0.000000000003
		*MTMR6* gene	0.830 (0.755, 0.912)	0.0001
		*ACOT2* gene	1.175 (1.083, 1.274)	0.00009
		*PGAP3* gene	1.187 (1.102, 1.278)	0.000005
		*STARD3* gene	1.697 (1.318, 2.186)	0.00004
		*GSDMB* gene	1.718 (1.319, 2.238)	0.00005
		*ADAM33* gene	1.323 (1.145, 1.529)	0.0001
Zhong et al. 2024 [28]	INTERVAL/EPIC‐Norfolk PanScan PanC4	Blood metabolites (X:12798, X:11787, X:11308, and X:19141)	FDR < 0.05	
Zou et al. 2024 [16]	Publicly available genetic dataset	CD4^+^ CD8dim %leukocyte	0.852 (0.729–0.995)	0.0430
		HLA DR + CD4^+^ in TBNK (T‐cell, B‐cell, natural killer cell)AC	0.933 (0.883–0.986)	
		CD28 on CD45RA− CD4 non‐Treg	1.155 (1.028–1.297)	0.016
		CD25 on activated Treg in Treg cells, among others	1.180 (1.014–1.374)	0.032
Min et al. 2023 [20]	IIBDGC	Inflammatory bowel disease ulcerative colitis	0.951 (0.648–1.397	0.799
			0.910 (0.753–1.101)	0.332
			0.946 (0.830–1.079)	0.409
			0.902 (0.682–1.194)	0.474
		Inflammatory bowel disease Crohn's disease	1.338 (1.064–1.683)	0.015
			1.120 (0.970–1.292)	0.119
			1.111 (1.015–1.213)	0.022
			1.353 (1.100–1.662)	0.005

*Note:* (OR > 1): potential risk factors. (OR < 1): protective benefits.

### 2.3 Association of Pancreatic Cancer With Non‐Malignant Phenotypes

A two‐way MR analysis was conducted to investigate the causal effects of obesity, measured by body mass index (BMI) and abdominal obesity using waist‐to‐hip ratio adjusted for BMI (WHRadjBMI), on pancreatic cancer. BMI is a widely used measure that assesses body fat based on an individual's weight and height. It provides a general indication of body composition but does not account for the distribution of fat within the body. In contrast, WHRadjBMI specifically focuses on abdominal fat distribution, which is often more closely associated with metabolic health and disease risk. The analysis involved calculating weighted polygenic scores (PGS) for both BMI and WHRadjBMI. The PGS for BMI was significantly associated with pancreatic cancer (OR = 1.0804 [1.025–1.14], *p* = 0.0037), even after correcting for multiple testing. However, this association was attenuated after adjusting for type 2 diabetes (OR = 1.073 [1.018–1.13], *p* = 0.00904). The association between the PGS for WHRadjBMI and pancreatic cancer was marginally significant (OR = 1.047 [0.99–1.104], *p* = 0.086). This association was significantly reduced after adjusting for type 2 diabetes (OR = 1.039 [0.99–1.097], *p* = 0.14). The MR analyses suggested that abdominal adiposity, as measured by WHRadjBMI, is likely a more significant causal risk factor for pancreatic cancer (OR = 1.00095 [1.00011–1.0018], *p* = 0.027) than total adiposity measured by BMI. Overall, the abdominal fat index was identified as a more critical risk factor for pancreatic cancer compared to total obesity, which may be influenced by type 2 diabetes [26]. Our analysis indicates that WHRadjBMI may be a more significant causal risk factor for pancreatic cancer than BMI due to its stronger association with metabolic conditions such as type 2 diabetes, which is known to influence pancreatic cancer development. This stronger link suggests that visceral fat may produce higher levels of inflammatory cytokines and hormones that can influence cancer development.

In another study on 8803 patients with PDAC and 67 523 controls, Tan et al. investigated the relationship between genetically determined gene expression in normal pancreatic tissue and the risk of PDAC. MR was used to analyze the causal relationships between PDAC, type 2 diabetes, and venous thromboembolism (VTE). Sixteen genes were found to be associated with the risk of pancreatic PDAC at a FDR of less than 0.10. This group included six novel genes (*PPIP5K2*, *TFR2*, *HNF4G*, *LRRC10B*, *PRC1*, and *FBXL20*) not previously linked to PDAC risk. Additionally, 10 genes that have been reported in earlier studies (*INHBA*, *SMC2*, *ABO*, *PDX1*, *MTMR6*, *ACOT2*, *PGAP3*, *STARD3*, *GSDMB*, and *ADAM33*) were also identified. The study also revealed that specific genetic variants linked to type two diabetes are associated with an elevated risk of pancreatic cancer. Furthermore, the genetic susceptibility to pancreatic cancer appears to extend to thrombotic events as well. *HNF4G* and *PDX1* may contribute to the development of diabetes associated with PDAC, while the *ABO* gene may mediate the causal relationship between VTE and PDAC [14].

Previous observational studies have shown an association between intra‐pancreatic fat deposition (IPFD) and PDAC, however, the causal relationship remains unclear. To elucidate this causality, a prospective observational study was conducted, incorporating IPFD assessment using magnetic resonance imaging (MRI) and genetic analyses from the UK Biobank. This study found that high levels of IPFD (> 10%) were associated with an increased risk of PDAC (adjusted hazard ratio [HR]: 3.35, [95% CI]: 1.60–7.00). An IVW Mendelian randomization study was also performed, utilizing nine genetic variants associated with IPFD (*p* < 5 × 10–8). Eight of these variants revealed a genetic association between IPFD and PDAC (OR: 2.46, 1.38–4.40) in the PanScan I‐III and Pancreatic Cancer Case–Control Consortium (PanC4) dataset. This study provides evidence suggesting a potential causal role of IPFD in the pathogenesis of PDAC. Consequently, reducing IPFD may reduce the risk of developing PDAC [15].

However, the relationship between nonalcoholic fatty liver disease (NAFLD) and pancreatic cancer susceptibility is complex and marked by conflicting data. MR analyses of 77 single nucleotide polymorphisms (SNPs) associated with NAFLD, defined by chronically elevated serum alanine aminotransferase levels (*p* < 5 × 10–8), and pancreatic cancer risk were conducted using data from the Cohort Consortium (PanScan) and the Pancreatic Cancer Case–Control Consortium (PanC4) GWAS. These analyses revealed that only one NAFLD‐associated SNP, ABO‐rs687621 (*p*‐value = 1.15 × 10–17 for PanScan and 1.31 × 10–13 for PanC4), was associated with pancreatic cancer. Therefore, genetic predisposition to NAFLD does not appear to be directly associated with the risk of pancreatic cancer (PanScan, IVW OR, 1.04, 0.88–1.22; MR‐Egger OR, 0.89, 0.65–1.21; PanC4, IVW OR, 1.07, 0.90–1.27; MR‐Egger OR, 0.93, 0.67–1.28). Given the close link between NAFLD and various metabolic conditions, any observed association between NAFLD and pancreatic cancer may be influenced by underlying metabolic disorders such as obesity, diabetes, or metabolic syndrome, rather than a direct causal relationship between NAFLD and pancreatic cancer [27].

### 2.4 Impact of Blood Metabolites on Pancreatic Cancer Risk

In a recent two‐sample MR study by Zhong et al. the potential causal effects of plasma metabolites on PDAC risk was investigated.

Genetic instruments were identified for a total of 506 metabolites from a comprehensive GWAS from the INTERVAL and EPIC‐Norfolk cohorts. Additionally, another set of genetic instruments was created for 483 metabolites based on an independent GWAS from the Canadian Longitudinal Study on Aging (CLSA) cohort. Data from the Pancreatic Cancer Cohort Consortium (PanScan) and the Pancreatic Cancer Case–Control Consortium (PanC4) were analyzed. The relationship between metabolites and PDAC risk was evaluated using the IVW method. Furthermore, a phenome‐wide Mendelian randomization (Phe‐MR) analysis was conducted to assess potential side effects of targeting the identified metabolites for PDAC intervention.

A total of 44 unique metabolites were found to be significantly associated with the risk of pancreatic ductal adenocarcinoma (PDAC). Among these, four metabolites (X: 12798, X: 11787, X: 11308, and X: 19141) demonstrated replication across instruments developed from both cohorts. These findings underscore the importance of novel blood metabolites in relation to PDAC risk, potentially guiding future research into metabolic characteristics and their roles in assessing PDAC risk [28].

### 2.5 Causal Effects of Immune Cells on Pancreatic Cancer

The research conducted by Zou et al. employed a two‐sample MR approach to explore the causal relationship between immune cells and the risk of developing pancreatic cancer. An analysis of 731 immunophenotypes derived from a publicly available genetic dataset revealed 24 immunophenotypes that are suggestively associated with pancreatic cancer (Table 1). Notably, certain phenotypes, such as the percentage of CD4+ CD8dim leukocytes (OR = 0.852, 0.729–0.995, *p* = 0.0430) and HLA DR+ CD4+ activated T cells (OR = 0.933, 0.883–0.986) within TBNK cells (T cells, B cells, and natural killer cells), demonstrated an inverse relationship with pancreatic cancer risk. Conversely, other phenotypes, including CD28 on CD45RA− CD4 non‐Treg (OR = 1.155, 1.028–1.297, *p* = 0.016) and CD25 on activated Treg (OR = 1.180, 1.014–1.374, *p* = 0.032) within T regulatory cells, exhibited a positive correlation with pancreatic cancer risk [16]. The findings underscore the possible involvement of immune cells in the development of pancreatic cancer, indicating that immune modulation may serve as a promising target for both prevention and treatment strategies.

### 2.6 Causal Effect of Inflammatory Bowel Disease on Pancreatic Cancer

A two‐sample MR analysis was conducted to investigate the causal relationship between inflammatory bowel disease (IBD) and the incidence of pancreatic cancer. The analysis utilized GWAS data from East Asian and European populations. Wald ratio, IVW, MR‐Egger, weighted median, and weighted mode were used to assess the causal effect of IBD on pancreatic cancer.

In the East Asian population, no significant causal relationship was found between IBD and pancreatic cancer. The results revealed that Crohn's disease, a type of IBD, had a significant causal effect on pancreatic cancer risk by 11.1% using the IVW method (*p* = 0.022), 33.8% using the MR‐Egger method (*p* = 0.015), and 35.3% using the weighted model (*p* = 0.005).

In contrast, ulcerative colitis, another form of IBD, did not show any statistically significant causal effect on pancreatic cancer risk (*p* > 0.05), as detailed in Table 1. The pleiotropic test and leave‐one‐out analysis confirmed the validity and reliability of the two‐sample MR analyses.

These findings suggest that Crohn's disease, but not ulcerative colitis, may contribute to an increased risk of developing pancreatic cancer in the European population. However, no significant causal relationship between IBD and pancreatic cancer was observed in the East Asian population [20].

## 3 Discussion

Addressing the challenges of identifying high‐risk groups and discovering suitable biomarkers for pancreatic cancer, as well as enhancing treatment effectiveness, are key research priorities. Previous epidemiological studies have identified several biomarkers through GWAS, but the causal relationships remain largely undefined. Mendelian Randomization, a method utilizing genetic variants such as SNPs, offers a way to assess the causal effects of exposures while minimizing the impact of confounding factors [21].

The gut microbiome has become increasingly recognized as a critical factor in the development of various cancers, including pancreatic cancer. Research suggests that changes in the gut microbiome can affect cancer progression through multiple mechanisms [29, 30]. Recent studies suggest that the gut microbiome interacts with various risk factors for pancreatic cancer, such as obesity and diabetes, and can induce an inflammatory response. Furthermore, the gut microbiome influences the development and progression of pancreatic cancer by modulating inflammatory responses, immune cell infiltration, and other mechanisms, although the causal effects of these interactions require further investigation [9].

Abnormal intestinal microbiota may contribute to the development of pancreatic cancer by impacting local intestinal immunity, T‐cell growth, and immune system maturation. Microbial metabolites can lead to chronic inflammation, which is often exacerbated by unhealthy diets. Conversely, MR analysis has indicated that *Senegalimassilia*, known for its protective effects against hypertension and metabolic and inflammatory processes, may have an inhibitory effect on pancreatic cancer. *Ruminiclostridium 9* has also been shown to regulate lipid metabolism, reduces inflammation, enhances intestinal barrier function, and increases insulin sensitivity, thereby potentially reducing the development of obesity—a known risk factor for pancreatic cancer. The identification of *Senegalimassilia* as a protective factor, in contrast to *Odoribacter*, *Ruminiclostridium 9*, *Ruminococcaceae*, and *Streptococcus* as risk factors, highlights the dual role of the gut microbiota in influencing cancer risk [5].

The integration of findings from Li et al. further clarifies the genetic basis of gut microbiome interactions with pancreatic cancer. The discovery of genes such as MCM6 and RPS26, which are involved in these interactions, not only underscores the genetic susceptibility to microbiome‐related cancer pathways but also opens up possibilities for targeted therapies [10]. Future research should aim to elucidate the mechanisms by which these bacteria exert their effects, potentially leading to probiotic or dietary interventions that could modify gut microbiota composition to reduce the risk of PC. In addition, the role of the oral microbiome in pancreatic cancer risk is a critical area of investigation. The identification of *Fusobacterium* and Veillonellaceae F0422 as potential risk factors, alongside several protective genera, indicates that oral health may have significant implications for systemic diseases, including cancer [25]. The oral cavity acts as a reservoir for bacteria that can translocate to other parts of the body, and understanding this microbiome's role could lead to novel preventive measures [31]. These findings suggest a need to reassess oral health practices and their potential impact on pancreatic cancer risk. However, further studies are necessary to elucidate the causal pathways linking gut and oral microbiome composition with pancreatic cancer.

The relationship between non‐malignant phenotypes, such as obesity and metabolic disorders, and pancreatic cancer has been extensively documented [32]. MR analysis has revealed a significant association between BMI and pancreatic cancer, although this association is attenuated after adjusting for type two diabetes [26]. This finding underscores the complex interplay between metabolic health and cancer risk, suggesting that while obesity is a risk factor, its influence may be partially mediated through diabetes‐related mechanisms. Furthermore, a MR study demonstrated that IPFD has a causal role in increasing the risk of PDAC, potentially up to three times. This increased risk appears to be independent of general obesity, as indicated by BMI‐adjusted results from an observational study and further supported by a Mendelian randomization study that excluded genetic variants nominally associated with BMI [14, 15]. One possible mechanism linking IPFD and PDAC involves the increased production of cytokines and adipokines by adipocytes within the pancreas. These cytokines and adipokines can induce low‐grade chronic inflammation, suppress apoptosis, and promote cell proliferation and migration, all of which can contribute to the development or progression of cancer. IPFD may serve as a non‐invasive biomarker for PDAC risk, particularly for individuals already at high risk due to factors such as chronic pancreatitis, adult‐onset diabetes, inherited predisposing mutations, or family history. Notably, the results of the MR study suggest that reducing IPFD could lower the incidence of PDAC. Polygenic analyses and MR also indicate that metabolic syndrome, often caused by abdominal obesity, may be a risk factor for pancreatic cancer. Additionally, obesity‐related type two diabetes could be a contributing factor to the metabolic syndrome underlying the development of pancreatic cancer in obese individuals [26].

Regarding genetic susceptibility to NAFLD and its potential relationship with the risk of pancreatic cancer, in a two‐sample MR study, a causal relationship between genetic susceptibility to NAFLD and increased risk of pancreatic cancer has been shown. Specifically, the findings showed that individuals with a higher genetic predisposition to NAFLD were significantly at increased risk of developing pancreatic cancer. This study provided strong evidence of a causal relationship between genetic predisposition to NAFLD and pancreatic cancer risk, suggesting that interventions targeting NAFLD could be beneficial in reducing pancreatic cancer risk [27].

The causal link between IBD and pancreatic cancer, especially the elevated risk associated with Crohn's disease, highlights the significant role of chronic inflammation in cancer development. The differing impacts of various types of IBD on pancreatic cancer risk indicate that the underlying mechanisms may vary. These findings emphasize the importance of increased surveillance for pancreatic cancer in patients with IBD [20].

Additionally, MR findings revealed that high stability immunophenotypes, characterized by the absence of horizontal pleiotropy and heterogeneity, exert an inhibitory effect on pancreatic cancer. However, 7 out of the 16 immunophenotypes that positively influenced pancreatic cancer belonged to the regulatory T cell subset. This discovery underscores the crucial role of Tregs, particularly the CD25 phenotype, which exhibited the strongest positive correlation with pancreatic cancer. The inverse relationship between certain immune cell types, such as CD4+ CD8dim leukocytes, and pancreatic cancer risk suggests a protective immune response that may be compromised during cancer development. Conversely, the positive correlation of other immune cell types with cancer risk indicates a complex immune landscape that may facilitate tumor progression [16].

Finally, the potential for metabolomic profiling to serve as a biomarker for pancreatic cancer risk is highly promising. If validated, these biomarkers could significantly enhance early detection efforts, enabling timely interventions in high‐risk populations. The identification of 44 unique metabolites associated with PDAC risk offers a new perspective on the metabolic dysregulation that may precede cancer development [28]. Further research is necessary to elucidate the biological roles and mechanisms of action of these metabolites.

The insights derived from MR studies on pancreatic cancer underscore the necessity for a multifaceted approach to cancer prevention and management. The intricate interplay between genetic, microbial, metabolic, and immune factors demands interdisciplinary collaboration among researchers, clinicians, and public health professionals. Future research should prioritize the integration of multi‐omics approaches, combining genomics, metabolomics, and microbiome analyses to achieve a comprehensive understanding of pancreatic cancer etiology. Additionally, exploring lifestyle interventions such as dietary modifications and physical activity should be emphasized as potential strategies for reducing cancer risk. In clinical practice, these findings highlight the importance of personalized medicine, considering individual genetic and metabolic profiles. Screening and preventive strategies should be tailored to high‐risk populations, including those with obesity, diabetes, and chronic inflammatory conditions.

While MR studies provide strong evidence for potential causal relationships and help reduce confounding factors, they cannot definitively establish causality. It is important to note that although our findings suggest causal links, they are based on observational data and do not constitute definitive proof of causality. There are several limitations associated with this type of study, including: (a) the inherent limitations of MRI studies, which may involve potential violations of underlying assumptions, (b) the possibility of residual confounding due to unmeasured variables that may affect both the exposure and the outcome, and (c) the generalizability of our findings, which may be restricted to specific populations or ethnic groups represented in the analyzed studies.

## 4 Conclusion

The studies demonstrated that MR can be valuable for identifying new biomarkers for screening and predicting pancreatic cancer, as well as for determining therapeutic targets by examining combinations that target specific proteins. The use of MR studies to investigate causal exposures in pancreatic cancer has yielded significant insights into the multifactorial nature of this disease. These studies have shed light on the roles of the gut and oral microbiomes, non‐malignant phenotypes, blood metabolites, immune dynamics, and chronic inflammation. This knowledge opens up new avenues for innovative prevention and treatment strategies. Ongoing research in this area is crucial for translating these findings into clinical practice, ultimately enhancing outcomes for individuals at risk of pancreatic cancer. To strengthen causal inferences and clarify the relationships between exposures and outcomes, additional studies—such as longitudinal studies, clinical trials, or mechanistic research—are often required. These complementary approaches can provide more robust evidence to support or challenge causal claims derived from MR analyses.

## Conflicts of Interest

The authors declare no conflicts of interest.
